# Interim Estimated Effectiveness of 2025-2026 COVID-19 Vaccines in Adults Using a Test-Negative Design

**DOI:** 10.1001/jamanetworkopen.2026.25152

**Published:** 2026-06-23

**Authors:** Ryan E. Wiegand, Sean Chickery, Duck-Hye Yang, Sarah W. Ball, Malini B. DeSilva, Kristin Dascomb, Stephanie A. Irving, Karthik Natarajan, Nicola P. Klein, Shaun J. Grannis, Toan C. Ong, Elizabeth A. K. Rowley, Adam Yates, Yan Zhuang, Sarah Wilson, Charlene E. McEvoy, Inih J. Essien, Omobosola O. Akinsete, Allison L. Naleway, Padma Koppolu, Ousseny Zerbo, John R. Hansen, Karen B. Jacobson, Lawrence Block, Brian E. Dixon, Thomas Duszynski, Colin Rogerson, Michelle A. Barron, Catia Chavez, Josephine Mak, Allison Avrich Ciesla, Monica Godfrey, Amber Kautz, Morgan Najdowski, Ruth Link-Gelles, Jennifer DeCuir, Amanda B. Payne

**Affiliations:** 1 National Center for Immunization and Respiratory Diseases, Centers for Disease Control and Prevention, Atlanta, Georgia; 2 Westat, Bethesda, Maryland; 3 HealthPartners Institute, Minneapolis, Minnesota; 4 Division of Infectious Diseases and Clinical Epidemiology, Intermountain Health, Salt Lake City, Utah; 5 Kaiser Permanente Center for Health Research, Portland, Oregon; 6 Department of Biomedical Informatics, Columbia University Irving Medical Center, New York, New York; 7 Kaiser Permanente Vaccine Study Center, Kaiser Permanente Northern California Division of Research, Oakland; 8 Center for Biomedical Informatics, Regenstrief Institute, Indianapolis, Indiana; 9 Department of Family Medicine, School of Medicine, Indiana University, Indianapolis; 10 School of Medicine, University of Colorado Anschutz Medical Campus, Aurora; 11 Department of Health Policy and Management, Richard M. Fairbanks School of Public Health, Indiana University, Indianapolis; 12 Department of Epidemiology, Richard M. Fairbanks School of Public Health, Indiana University, Indianapolis; 13 Department of Pediatrics, School of Medicine, Indiana University, Indianapolis; 14 Eagle Health Analytics, San Antonio, Texas; 15 General Dynamics Information Technology, Atlanta, Georgia; 16 US Public Health Service Commissioned Corps, Rockville, Maryland

## Abstract

**Importance:**

The 2025-2026 COVID-19 vaccine, targeting JN.1 and JN.1-derived sublineages, became available in the US in September 2025.

**Objective:**

To assess the estimated interim effectiveness of 2025-2026 COVID-19 vaccines against medically attended COVID-19 among immunocompetent adults aged 18 years or older in the US.

**Design, Setting, and Participants:**

This case-control study used a test-negative design to investigate patient encounters captured in the Virtual SARS-CoV-2, Influenza, and Other Respiratory Viruses Network, an electronic medical record–based network of health care systems (253 emergency departments/urgent cares [ED/UCs] and 179 hospitals in 7 states) from September 3, 2025, to December 31, 2025. Patient encounters with COVID-19–like illness and a molecular or antigen SARS-CoV-2 test 10 days before to 3 days after the encounter date were included.

**Exposure:**

2025-2026 COVID-19 vaccination regardless of prior COVID-19 vaccination.

**Main Outcomes and Measures:**

The main outcomes were COVID-19–associated ED/UC encounters and COVID-19–associated hospitalizations. Cases were defined as encounters with a positive molecular or antigen SARS-CoV-2 test and controls as encounters with a negative molecular SARS-CoV-2 test. The odds of 2025-2026 COVID-19 vaccination among cases and controls, adjusting for confounders, were compared and used to estimate vaccine effectiveness (VE) as (1 − adjusted odds ratio) × 100%.

**Results:**

In 85 725 ED/UC encounters among adults aged 18 years and older (51 841 [60%] aged 18-64 years; 51 775 female [60%]), 206 of 3941 cases (5%) and 9453 of 81 784 controls (12%) received a 2025-2026 COVID-19 vaccination. Estimated VE against COVID-19–associated ED/UC encounters was 50% (95% CI, 42%-57%; median [IQR] time since 2025-2026 COVID-19 vaccine dose receipt, 47 [27-69] days). In 26 073 hospitalizations with a COVID-19–like illness (17 530 [67%] aged ≥65 years; 13 985 female [54%]), 60 of 1022 cases (6%) received a 2025-2026 COVID-19 vaccination compared with 3080 of 25 051 controls (12%). Estimated VE against COVID-19–associated hospitalization was 55% (95% CI, 41%-66%; median [IQR] time since 2025-2026 COVID-19 vaccine dose receipt, 46 [26-68] days). Among patients aged 65 years or older, estimated VE against ED/UC encounters was 48% (95% CI, 37%-56%; median [IQR] time since dose receipt, 48 [27-69] days; 33 884 encounters) and against hospitalization was 53% (95% CI, 37%-65%; median [IQR] time since dose receipt, 46 [26-69] days; 17 530 hospitalizations).

**Conclusions and Relevance:**

In this study, receipt of 2025-2026 COVID-19 vaccination was associated with additional protection beyond existing immunity in adults against medically attended COVID-19, including ED/UC encounters and hospitalizations, compared with no receipt of a 2025-2026 vaccine dose. These findings suggest that adults can reduce their likelihood of severe COVID-19–associated outcomes by obtaining a 2025-2026 COVID-19 vaccination.

## Key Points

**Question:**

What was the estimated effectiveness of 2025-2026 COVID-19 vaccines against medically attended COVID-19 among adults aged 18 years or older in the US from September to December 2025?

**Findings:**

In this case-control study of 85 725 emergency department and urgent care (ED/UC) encounters and 26 073 hospitalizations in immunocompetent adults aged 18 years or older with a COVID-19–like illness, estimated effectiveness of 2025-2026 COVID-19 vaccination was 50% against COVID-19–associated ED/UC encounters and 55% against COVID-19–associated hospitalization, compared with not receiving a 2025-2026 vaccine dose.

**Meaning:**

This study found that 2025-2026 COVID-19 vaccines were associated with additional protection against medically attended COVID-19 beyond individuals’ existing immunity, suggesting that adults can reduce their likelihood of severe COVID-19–associated outcomes by obtaining a 2025-2026 COVID-19 vaccination.

## Teaser

This case-control study estimates vaccine effectiveness of 2025-2026 COVID-19 vaccines among adults with COVID-19–associated emergency department or urgent care encounters and COVID-19–associated hospitalizations.

## Introduction

From October 1, 2024, to September 27, 2025, COVID-19 was associated with an estimated 390 000 to 550 000 hospitalizations in the US^1^; the highest COVID-19–associated hospitalization rate (406 per 100 000 population) was reported among adults aged 65 years and older.^2^ In May 2025, the US Food and Drug Administration (FDA) recommended that 2025-2026 COVID-19 vaccines should be monovalent JN.1 lineage-based, preferentially using the LP.8.1 strain, to more closely match currently circulating SARS-CoV-2 viruses.^3^ On August 27, 2025, the FDA approved monovalent 2025-2026 COVID-19 vaccines by Moderna^4^ and Pfizer-BioNTech^5^ based on the SARS-CoV-2 Omicron LP.8.1 strain and approved a monovalent 2025-2026 COVID-19 vaccine by Novavax^6^ based on the SARS-CoV-2 Omicron JN.1 lineage.

Continual monitoring of vaccine effectiveness (VE), particularly when new vaccine formulations are introduced, can provide important information to guide patient and clinician decision-making. This analysis estimated 2025-2026 COVID-19 VE against COVID-19–associated emergency department and urgent care (ED/UC) encounters and COVID-19–associated hospitalizations from September 3, 2025, to December 31, 2025, among immunocompetent adults aged 18 years or older via a test-negative design.

## Methods

This case-control study was reviewed by the Centers for Disease Control and Prevention (CDC), deemed not research, and was conducted consistent with applicable federal law and CDC. This activity was reviewed and approved as a research activity by 1 Virtual SARS-CoV-2, Influenza, and Other Respiratory Viruses Network (VISION) site (Kaiser Permanente Northern California). This study presented minimal risk to participants because there was no patient interaction or intervention; therefore, a waiver of informed consent was granted. This study followed the Strengthening the Reporting of Observational Studies in Epidemiology (STROBE) reporting guideline.^9^

### Data Source

The test-negative study design and methods of VISION have been previously reported.^7^ VISION is a multisite, electronic health record–based network. The 5 VISION sites contributing data to this analysis included 253 ED/UCs and 179 hospitals in 7 states: HealthPartners (Minnesota and Wisconsin), Kaiser Permanente Northern California (California), Kaiser Permanente Northwest (Oregon and Washington), Regenstrief Institute (Indiana), and University of Colorado (Colorado).

### Study Design

The objective of the study was to estimate VE against ED/UC encounters and inpatient hospitalization among persons with a COVID-19–like illness using a test-negative design. Eligible encounters were ED/UC encounters or hospital admissions with COVID-19–like illness based on *International Statistical Classification of Diseases and Related Health Problems, Tenth Revision (ICD-10) *discharge diagnosis codes (eTable 1 in Supplement 1) among immunocompetent adults aged 18 years or older who received molecular testing (eg, real-time reverse transcription-polymerase chain reaction) or antigen testing for SARS-CoV-2 during the 10 days before or 72 hours after the ED/UC encounter or hospital admission. COVID-19 vaccination history was ascertained from jurisdictional immunization information systems, electronic health records, and, at some sites, medical claims data. Doses administered at pharmacies should have been reported to immunization information systems because national pharmacy chains were required to establish bidirectional linkage with immunization information systems early in the COVID-19 pandemic.

Case patients were persons with a COVID-19–like illness who received a positive SARS-CoV-2 molecular or antigen test result, and control patients were those who received a negative SARS-CoV-2 molecular test result. Vaccination was defined as receipt of a 2025-2026 COVID-19 vaccine 7 or more days before encounter index date. The index date was the date of respiratory specimen collection for the most recent positive or negative SARS-CoV-2 test or the encounter, whichever came first. ED/UC encounters 7 or fewer days or hospitalizations 30 or fewer days from the index date were collapsed into the earliest index date. For patients with multiple tests during these periods, priority was (1) a positive over any negative result, (2) the test closest to the encounter date, and (3) the test type in the following order: viral culture, molecular assay, rapid molecular assay, rapid antigen, fluorescent antibody, and serology.

Encounters were excluded among patients who (1) received a COVID-19 vaccine less than 7 days before their eligible ED/UC encounter or hospitalization, (2) received a 2025-2026 COVID-19 vaccine dose less than 2 months after receiving a previous COVID-19 vaccine dose, (3) received more than one 2025-2026 COVID-19 vaccine dose, or (4) were immunocompromised, based on *ICD-10* discharge codes (eTable 2 in Supplement 1). COVID-19 case patients who were coinfected with influenza viruses or respiratory syncytial virus at the time of their COVID-19–like illness encounter were also excluded. Because of potential confounding from correlated vaccination behaviors, control patients with positive or indeterminant test results for influenza viruses (age ≥18 years) or respiratory syncytial virus (age ≥60 years) were excluded.^8^

### Statistical Analysis

Adjusted odds ratios (aORs) and 95% CIs were estimated via multivariable logistic regression. The aORs compared patients who received a 2025-2026 COVID-19 vaccine dose with patients who did not among case patients and control patients, irrespective of previous COVID-19 vaccination, and were adjusted a priori for age, sex, race and ethnicity from the electronic health record, calendar time computed from the encounter date, and geographic region based on the location of the health system. Age and calendar time were included as natural cubic splines. Race and ethnicity included non-Hispanic Black or African American, Hispanic or Latino (any race), non-Hispanic White, non-Hispanic other race (American Indian or Alaska Native, Asian, Native Hawaiian or Other Pacific Islander, Middle Eastern or North African, races not otherwise listed, and multiple races), and unknown (no race or ethnicity data in the electronic health record); race and ethnicity were included to control for confounding and assess the generalizability of results across diverse populations. VE was calculated as (1 − aOR) × 100%. VE was estimated against ED/UC encounters and hospitalization separately for immunocompetent adults aged 18 years and older and 65 years and older. Limited statistical power precluded further analyses. Analyses were conducted using R software version 4.5.0 (R Project for Statistical Computing). Statistical significance was defined as 95% CI that did not cross the null.

## Results

### 2025-2026 COVID-19 Estimated VE Against COVID-19–Associated ED/UC Encounters

A total of 85 725 ED/UC encounters among immunocompetent adults aged 18 years or older with a COVID-19–like illness were analyzed, including 3941 case patients (5%) and 81 784 control patients (95%). Overall, 9659 patients (11%) had received a 2025-2026 COVID-19 vaccination before their encounter, with 206 (5%) among case patients and 9453 (12%) among control patients (Table 1). Among all encounters, 51 841 (60%) were immunocompetent adults aged 18 to 64 years and 51 775 (60%) were female. The majority of ED/UC encounters occurred among non-Hispanic White patients (51 766 encounters [60%]), followed by Hispanic or Latino patients (12 685 encounters [15%]), non-Hispanic Black or African American patients (10 487 encounters [12%]), and patients with other non-Hispanic race or ethnicity 9119 encounters [11%]). The largest number of ED/UC encounters occurred in December 2025 (26 735 encounters [31%]). Patients with ED/UC encounters had a median (IQR) of 0 (0-1) underlying medical condition categories.

**Table 1.** Characteristics of ED/UC Encounters and Hospitalizations Among Immunocompetent Adults With COVID-19–Like Illness, by COVID-19 Case Status—VISION, US, September-December 2025^a^

Characteristic	ED/UC encounters				Hospitalizations			
	Total, No. (%) (n = 85 725)	SARS-CoV-2 status, No. (%)		SMD	Total, No. (%) (n = 26 073)	SARS-CoV-2 status, No. (%)		SMD
		Positive (cases) [n = 3941]	Negative (controls) [n = 81 784]			Positive (cases) [n = 1022]	Negative (controls) [n = 25 051]	
2025-2026 COVID-19 vaccination status								
No 2025-2026 dose received^b^	76 066 (89)	3735 (95)	72 331 (88)	0.23	22 933 (88)	962 (94)	21 971 (88)	0.23
2025-2026 dose received 7-118 d earlier	9659 (11)	206 (5)	9453 (12)		3140 (12)	60 (6)	3080 (12)	
Age, median (IQR), y	56 (35-74)	57 (36-75)	56 (35-74)	0.03	72 (60-82)	77 (66-85)	72 (60-82)	0.28
Age group, y^c^								
18-64	51 841 (60)	2356 (60)	49 485 (61)	0.02	8543 (33)	226 (22)	8317 (33)	0.25
≥65	33 884 (40)	1585 (40)	32 299 (39)		17 530 (67)	796 (78)	16 734 (67)	
Sex								
Female	51 775 (60)	2383 (60)	49 392 (60)	<0.01	13 985 (54)	539 (53)	13 446 (54)	0.02
Male	33 950 (40)	1558 (40)	32 392 (40)		12 088 (46)	483 (47)	11 605 (46)	
Race and ethnicity								
Black or African American, non-Hispanic	10 487 (12)	410 (10)	10 077 (12)	0.11	2805 (11)	79 (8)	2726 (11)	0.15
Hispanic or Latino, any race	12 685 (15)	574 (15)	12 111 (15)		2355 (9)	81 (8)	2274 (9)	
White, non-Hispanic	51 766 (60)	2527 (64)	49 239 (60)		18 205 (70)	775 (76)	17 430 (70)	
Other, non-Hispanic^d^	9119 (11)	338 (9)	8781 (11)		2404 (9)	78 (8)	2326 (9)	
Unknown^e^	1668 (2)	92 (2)	1576 (2)		304 (1)	9 (1)	295 (1)	
No. of organ systems associated with a chronic medical condition, median (IQR)^f^	0 (0-1)	0 (0-0)	0 (0-1)	0.10	4 (1-7)	4 (1-7)	4 (1-7)	0.09
Month of COVID-19-associated ED/UC encounter								
September 2025	19 007 (22)	1542 (39)	17 465 (21)	0.42	5831 (22)	384 (38)	5447 (22)	0.40
October 2025	19 532 (23)	729 (18)	18 803 (23)		6108 (23)	183 (18)	5925 (24)	
November 2025	20 451 (24)	581 (15)	19 870 (24)		6484 (25)	155 (15)	6329 (25)	
December 2025	26 735 (31)	1089 (28)	25 646 (31)		7650 (29)	300 (29)	7350 (29)	

Abbreviations: ED/UC, emergency department/urgent care; SMD, standardized mean difference; VISION, Virtual SARS-CoV-2, Influenza, and Other Respiratory Viruses Network.

^a^ Centers for Disease Control and Prevention–funded VISION sites that contributed data for this analysis were HealthPartners (Minnesota and Wisconsin), Kaiser Permanente Northern California (California), Kaiser Permanente Northwest (Oregon and Washington), Regenstrief Institute (Indiana), and University of Colorado (Colorado).

^b^ Included all eligible persons who did not receive a 2025-2026 COVID-19 vaccine dose, regardless of number of previous doses (if any) received.

^c^ Among adults aged 65 years or older with an ED/UC encounter, 149 of 1585 case-patients (9%) and 6758 of 32 299 of control patients (21%) received a 2025-2026 COVID-19 vaccination. Among hospitalized adults aged 65 years and older, 56 of 796 case-patients (7%) and 2662 of 16 734 of control patients (16%) received a 2025-2026 COVID-19 vaccination.

^d^ Persons reporting non-Hispanic ethnicity and any of the following for race: American Indian or Alaska Native, Asian, Native Hawaiian or Other Pacific Islander, Middle Eastern or North African, races not otherwise listed, and multiple races. Because of small numbers, these categories were combined.

^e^ Persons with no race or ethnicity data in their electronic heath record.

^f^ Underlying medical condition categories included pulmonary, cardiovascular, cerebrovascular, neurologic or musculoskeletal, hematologic, endocrine, kidney, and gastrointestinal.

Estimated VE of 2025-2026 COVID-19 vaccination against COVID-19-associated ED/UC encounters among all adults aged 18 years or older was 50% (95% CI, 42%-57%; median [IQR] time since 2025-2026 COVID-19 vaccine dose receipt, 47 [27-69] days) (Table 2). Among 33 884 adults aged 65 years or older, estimated VE against COVID-19–associated ED/UC encounters was 48% (95% CI, 37%-56%; median [IQR] time since 2025-2026 COVID-19 vaccine dose receipt, 48 [27-69] days).

**Table 2.** Estimated Effectiveness of 2025-2026 COVID-19 Vaccination Against COVID-19–Associated Emergency Department and Urgent Care Encounters Among Immunocompetent Adults, by Age Group and Vaccination Status—VISION, US, September-December 2025

Age group and COVID-19 vaccination status	Postitive SARS-CoV-2 status (cases), No./total No. (%)	Time since dose receipt median (IQR), d	Estimated vaccine effectiveness, % (95% CI)^a^	
			Unadjusted	Adjusted
≥18 y				
No 2025-2026 dose^b^	3735/76 066 (5)	1220 (415-1468)	Reference	Reference
Received 2025-2026 dose 7-118 d earlier	206/9659 (2)	47 (27-69)	58 (51-63)	50 (42-57)
≥65 y				
No 2025-2026 dose^b^	1436/26 977 (5)	720 (355-1390)	Reference	Reference
Received 2025-2026 dose 7-118 d earlier	149/6907 (2)	48 (27-69)	61 (53-67)	48 (37-56)

Abbreviation: VISION, Virtual SARS-CoV-2, Influenza, and Other Respiratory Viruses Network.

^a^ Vaccine effectiveness was calculated by comparing the odds of 2025-2026 COVID-19 vaccination in case patients and control patients using the equation (1 – adjusted odds ratio) × 100%. Odds ratios were estimated using multivariable logistic regression. Unadjusted estimates used a model with vaccination as the only covariate, whereas adjusted estimates were from models that also included age, sex, race and ethnicity, calendar day, and geographic region.

^b^ Included all eligible persons who did not receive a 2025-2026 COVID-19 vaccine dose, regardless of number of previous COVID-19 vaccine doses (if any) received.

### 2025-2026 COVID-19 Estimated VE Against COVID-19–Associated Hospitalization

A total of 26 073 immunocompetent adults aged 18 years or older were hospitalized with COVID-19–like illness, including 1022 case patients (4%) and 25 051 control patients (96%). Overall, 3140 patients (12%) had received 2025-2026 COVID-19 vaccination before their hospitalization, including 60 case patients (6%) and 3080 control patients (12%) (Table 1). Among all hospitalizations, 17 530 (67%) were immunocompetent adults aged 65 years or older and 13 985 (54%) were female. More than two-thirds of hospitalizations (18 205 hospitalizations [70%]) occurred among non-Hispanic White patients, followed by non-Hispanic Black or African American patients (2805 hospitalizations [11%]), Hispanic or Latino patients (2355 hospitalizations [9%]), and patients with other non-Hispanic race (2404 hospitalizations [9%]). The largest number of encounters occurred in December 2025 (7650 hospitalizations [29%]). Hospitalized patients had a median (IQR) of 4 (1-7) underlying medical condition categories.

Estimated VE of a 2025-2026 COVID-19 vaccine dose against COVID-19–associated hospitalization among immunocompetent adults aged 18 years or older was 55% (95% CI, 41%-66%; median [IQR] time since 2025-2026 COVID-19 vaccine dose receipt, 46 [26-68] days) (Table 3). Among 17 530 immunocompetent adults aged 65 years or older, estimated VE of 2025-2026 COVID-19 vaccination against COVID-19–associated hospitalization was 53% (95% CI, 37%-65%; median [IQR] time since 2025-2026 COVID-19 vaccine dose receipt, 46 [26-69] days).

**Table 3.** Estimated Effectiveness of 2025-2026 COVID-19 Vaccination Against COVID-19–Associated Hospitalization Among Immunocompetent Adults, by Age Group and Vaccination Status—VISION, US, September-December 2025

Age group and COVID-19 vaccination status	Positive SARS-CoV-2 status (cases), No./total No. (%)	Time since dose receipt, median (IQR), d	Estimated vaccine effectiveness, % (95% CI)^a^	
			Unadjusted	Adjusted
≥18 y				
No 2025-2026 dose^b^	962/22 933 (4)	1095 (390-1446)	Reference	Reference
Received 2025-2026 dose 7-114 d earlier	60/3140 (2)	46 (26-68)	56 (42-66)	55 (41-66)
≥65 y				
No 2025-2026 dose^b^	740/14 812 (5)	841 (371-1418)	Reference	Reference
Received 2025-2026 dose 7-114 d earlier	56/2718 (2)	46 (26-69)	60 (47-70)	53 (37-65)

Abbreviation: VISION, Virtual SARS-CoV-2, Influenza, and Other Respiratory Viruses Network.

^a^ Vaccine effectiveness was calculated by comparing the odds of 2025-2026 COVID-19 vaccination in case patients and control patients using the equation (1 – adjusted odds ratio) × 100%. Odds ratios were estimated using multivariable logistic regression. Unadjusted estimates used a model with vaccination as the only covariate, whereas adjusted estimates were from models that also included age, sex, race and ethnicity, calendar day, and geographic region.

^b^ Included all vaccination-eligible persons who did not receive a 2025-2026 COVID-19 vaccine dose, regardless of number of previous COVID-19 vaccine doses (if any) received.

## Discussion

In this case-control study with a test-negative design among immunocompetent adults aged 18 years or older, receipt of 2025-2026 COVID-19 vaccination from September to December 2025 provided protection against COVID-19–associated ED/UC encounters and hospitalization compared with not receiving a 2025-2026 COVID-19 vaccine dose. Moreover, the findings in this study demonstrate the added benefit of 2025-2026 COVID-19 vaccination irrespective of protection conferred by previous COVID-19 vaccination or SARS-CoV-2 infection.^10,11,12^ The test-negative design has been used extensively to evaluate effectiveness of respiratory virus vaccines and provides a convenient and efficient method to rapidly evaluate effectiveness of new vaccine products in real-world settings (eg, through EHR records).^13^

COVID-19–associated hospitalization rates during the early part of the 2025-2026 respiratory virus season (September to December) were lower than were those during previous years.^2^ However, as of February 28, 2026, the CDC estimated that 99 000 to 180 000 COVID-19–associated hospitalizations have occurred among persons of all ages in the US since October 1, 2025.^1^ Because SARS-CoV-2 viruses circulate year-round, persons who have not yet received a 2025-2026 COVID-19 vaccination can still benefit from receiving it. Previous season analyses indicated that COVID-19 vaccines provided protection against COVID-19-associated outpatient illness, hospitalization, and critical illness.^10,11,12^

During the analytic period, the primary circulating SARS-CoV-2 lineages were descendants of the Omicron JN.1 lineage, including XFG and NB.1.8.1.^14^ As of January 17, 2025, XFG was the predominant circulating lineage in the US; this lineage is antigenically related to the LP.8.1 strain and JN.1 lineage in the 2025-2026 COVID-19 vaccines.^15^ The pace and frequency with which new SARS-CoV-2 lineages have become predominant, as exemplified by the spread of the SARS-CoV-2 variant BA.3.2,^16^ underscores the value of ongoing monitoring of COVID-19 VE and genomic surveillance.

Protection conferred by SARS-CoV-2 infection wanes over time.^17^ However, SARS-CoV-2 circulation in the US increased just before the 2025-2026 COVID-19 vaccines became available^18^; because circulating SARS-CoV-2 lineages were predominantly descendants of the Omicron JN.1 lineage, this increased circulation might have led to higher population-level immunity against JN.1-lineage strains, and, as a consequence, lower estimated VE compared with VE in a population with less recent infection (eg, ≥1 years ago). Analyses did not account for previous SARS-CoV-2 infection or COVID-19 vaccination (eg, original monovalent, bivalent, 2023-2024, or 2024-2025 doses). VE should therefore be interpreted as the added benefit of 2025-2026 COVID-19 vaccination in a population with high levels of infection-induced immunity, vaccine-induced immunity, or both.

### Limitations

The findings in this study are subject to at least 4 limitations. First, case patients might have sought care at an ED/UC or hospital for reasons other than COVID-19, which could have resulted in lower VE estimates. Second, patient vaccination status might have been misclassified, which could affect VE estimates if misclassification differed between case patients and control patients. Third, the low rates of COVID-19-associated hospitalization^2^ and COVID-19 vaccination^19^ prevented estimation of VE in certain age groups and for certain outcomes. The combination of these factors led to reduced statistical power to estimate VE and precluded analyses of VE against critical illness (ie, intensive care unit admission, invasive mechanical ventilation, or death) for children aged 17 years or younger, adults with immunocompromise, adults aged 18 to 64 years, and time since vaccination. Fourth, residual confounding from unmeasured factors, such as behavioral modifications to prevent SARS-CoV-2 exposure and outpatient antiviral treatment for COVID-19, might remain.

## Conclusions

In this case-control study using a test-negative design, receipt of a 2025-2026 COVID-19 vaccine dose provided protection against COVID-19–associated ED/UC encounters and hospitalizations among immunocompetent adults. The CDC continues to monitor VE of COVID-19 vaccines in the context of changing virus dynamics and updated vaccine formulations. Adults can reduce their likelihood of severe COVID-19–associated outcomes by obtaining a 2025-2026 COVID-19 vaccination.

## Notes

Supplement 1. eTable 1. International Statistical Classification of Diseases and Related Health Problems, Tenth Revision (ICD-10) used to define COVID-19-like illness eTable 2. International Statistical Classification of Diseases and Related Health Problems, Tenth Revision (ICD-10) used to define underlying medical condition and immunocompromise status

Supplement 2. Data Sharing Statement
