# Supplementary material for: Interim Estimated Effectiveness of 2025-2026 COVID-19 Vaccines in Adults Using a Test-Negative Design
Source: JAMA Netw Open. 2026 Jun 23;9(6):e2625152. doi: 10.1001/jamanetworkopen.2026.25152 (PMC13291846; doi:10.1001/jamanetworkopen.2026.25152)
Supplement: Supplement 1. — eTable 1. International Statistical Classification of Diseases and Related Health Problems, Tenth Revision (ICD-10) used to define COVID-19-like illness eTable 2. International Statistical Classification of Diseases and Related Health Problems, Tenth Revision (ICD-10) used to define underlying medical condition and immunocompromise status [file jamanetwopen-e2625152-s001.pdf]

Supplemental Online Content

Wiegand RE, Chickery S, Yang DH, et al. Interim estimated vaccine effectiveness of 2025-2026 COVID-19 vaccines in adults using a test-negative design. *JAMA Netw. Open*. 2026;9(6):e2625152. doi:10.1001/ jamanetworkopen.2026.25152

eTable 1. *International Statistical Classification of Diseases and Related Health Problems, Tenth Revision (ICD-10)* used to define COVID-19-like illness

eTable 2. *International Statistical Classification of Diseases and Related Health Problems, Tenth Revision (ICD-10)* used to define underlying medical condition and immunocompromise status

This supplemental material has been provided by the authors to give readers additional information about their work.

© 2026 Wiegand RE et al. *JAMA Network Open*.

eTable 1. *International Statistical Classification of Diseases and Related Health Problems, Tenth Revision (ICD-10)* used to define COVID-19-like illness.

**Description of diagnosis**	***ICD-10* codes**
COVID-19 pneumonia	
Pneumonia due to SARS-associated coronavirus	J12.81
Pneumonia due to coronavirus disease 2019	J12.82
Influenza pneumonia	
Influenza due to identified novel influenza A virus with pneumonia	J09.X1
Influenza due to other identified influenza virus with pneumonia	J10.0*
Influenza due to unidentified influenza virus with pneumonia	J11.0*
Other viral pneumonia	J12.0, J12.1, J12.2, J12.3, J12.89, J12.9
Pneumonia due to *Streptococcus pneumoniae*	J13
Pneumonia due to *Hemophilus influenzae*	J14
Bacterial pneumonia, not elsewhere classified	J15.*
Pneumonia due to other infectious organisms, not elsewhere classified	J16.*
Pneumonia in diseases classified elsewhere	J17
Pneumonia, unspecified organism	J18.*
Influenza disease	J09.*, J10.1, J10.2, J10.8*, J11.1, J11.2, J11.8*,
Acute respiratory distress syndrome	J80
Chronic obstructive pulmonary disease with acute exacerbation	J44.1
Asthma acute exacerbation	J45.21, J45.22, J45.31, J45.32, J45.41, J45.42, J45.51, J45.52, J45.901, J45.902
Respiratory failure	
Acute respiratory failure	J96.0*
Acute and chronic respiratory failure	J96.2*
Respiratory arrest	R09.2
Respiratory failure, unspecified	J96.9*
Other acute lower respiratory tract infections	
Respiratory syncytial virus	B97.4
Acute bronchitis	J20.*
Acute bronchiolitis	J21.*
Unspecified acute lower respiratory infection	J22
Bronchitis, not specified as acute or chronic	J40
COPD with acute lower respiratory infection	J44.0
Simple and mucopurulent chronic bronchitis	J41.*
Unspecified chronic bronchitis	J42
Emphysema	J43.*
Bronchiectasis	J47.*
Abscess of lung and mediastinum	J85.*
Pyothorax	J86.*
Acute and chronic sinusitis	J01.*, J32.*
Acute upper respiratory tract infections	J00*, J02.*, J03.*, J04.*, J05*, J06.*
Acute respiratory illness signs and symptoms	
Hemoptysis	R04.2
Cough	R05, R05.1, R05.2, R05.4, R05.8, R05.9
Dyspnea unspecified	R06.00
Shortness of breath	R06.02
Acute respiratory distress	R06.03
Stridor	R06.1

© 2026 Wiegand RE et al. *JAMA Network Open*.

Wheezing	R06.2
Other abnormalities of breathing	R06.8
Apnea, not elsewhere classified	R06.81
Tachypnea, NEC	R06.82
Other abnormalities of breathing/ Other symptoms involving head & neck	R06.89
Chest pain on breathing/ painful respiration	R07.1
Asphyxia and hypoxemia	R09.0*
Pleurisy	R09.1
Respiratory arrest	R09.2
Abnormal sputum	R09.3
Other specified symptoms and signs involving the circulatory and respiratory systems	R09.8*
Acute febrile illness signs and symptoms	
Fever	R50.*
Fever presenting with conditions classified elsewhere	R50.81
Fever unspecified	R50.9
Chills (w/o fever)	R68.83
Febrile convulsions	R56.0*
Acute non-respiratory signs and symptoms	
Diarrhea	R19.7
Disturbance of smell and taste	R43.*
Headache	R51.9
Myalgia	M79.10, M79.18
Other malaise	R53.81
Other fatigue	R53.83
Altered level of consciousness / altered mental status	R41.82, R40.0, R40.1
Weakness	R53.1
Nausea and Vomiting	R11.0, R11.10, R11.11, R11.15, R11.2
Rash and other nonspecific skin eruption	R21*
Abdominal pain	R10.0, R10.1*, R10.2, R10.3*, R10.81*, R10.84, R10.85, R10.8A*, R10.A*, R10.9
Elevated C-reactive protein (CRP)	R79.82
Elevated sedimentation rate (ESR)	R70.0
Sepsis	R65.*
Shock, unspecified	R57.9
Viral and respiratory diseases complicating pregnancy, childbirth, and puerperium	O98.5*, O98.8*, O98.9*, O99.5*

*Includes all sub-codes.

© 2026 Wiegand RE et al. *JAMA Network Open*.

eTable 2. *International Statistical Classification of Diseases and Related Health Problems, Tenth Revision (ICD-10)* used to define underlying medical condition and immunocompromise status.

**Description of diagnosis**	***ICD-10* codes**
**Underlying Medical Conditions**	
Cardiovascular	
Heart failure	I50.*
Ischemic heart disease	I21.*, I22.*, I23.*, I24.*, I25.*
Hypertension	I10., I11.*, I13.*, I15.*
Other cardiovascular disease	I01.*, I02.0, I09.*, I27.*, I28.*, I31.*, I42.*, I43., I44.*, I46.*, I51.0, I51.1, I51.2, I51.3, I51.5, I51.7, I51.8*, I51.9, I52., I97.0, I97.1*, M31.0, M31.1*, M31.2, M31.4, M31.6, M31.7, M31.8, M31.9, Z95.*, Z98.61, I71.*, I72.*, I73.*, I74.*, I75.*, I79.*
Pulmonary embolism	I26.*
Heart valve disorder	I05.*, I06.*, I07.*, I08.*, I34.*, I35.*, I36.*, I37.*
Atrial fibrillation	I48.*
Congenital heart disease	I50.9, I42.9, Q20.*, Q21.*, Q22.*, Q23.*, Q24.*, Q25.*, Q26.*, Q27.0, Q27.3*, Q27.4, Q27.8, Q27.9, Q28.*, Q89.3, P29.30
Cerebrovascular	
Stroke	I60.*, I61.*, I63.*
Other cerebrovascular disease	I62.*, I68.*, I69.*
Endocrine	
Diabetes, Type 1	E10.*
Diabetes, Type 2	E11.*
Diabetes, Other	E08.*, E09.* E13.*
Other endocrine/metabolic condition	E00.*, E01.*, E03.*, E05.*, E06.*, E15., E16.*, E20.*, E21.*, E22.*, E23.*, E24.*, E25.*, E26.*, E27.*, E28.*, E29.*, E31.*, E32.*, E34.*, E70.*, E71.*, E72.*, E74.*, E75.2*, E76.*, E77.*, E79.*, E80.*, E83.*, E85.*, E88.02, E88.09, E88.1, E88.2, E88.3, E88.4*, E88.8*, E88.9
Lipid metabolism disorder	E78.*
Gastrointestinal	
Liver disorder	B18.*, I81, I85.*, K70.*, K71.*, K72.*, K73.*, K74.*, K75.*, K76.*, K77.
Chronic gastrointestinal disorder	K50.*, K51.*, K52.*
Hematologic	D55.*, D56.0, D56.1, D56.2, D56.4, D56.5, D56.8, D56.9, D57.0*, D57.1, D57.2*, D57.4*, D57.8*, D58.*, D59.*, D60.*, D61.*, D64.0, D64.1, D64.2, D64.3, D64.4, D64.8*, D65, D66, D67, D68.*
Musculoskeletal/Neurologic	
Dementia	F01.*, F02.*, F03.*, G30.*
Other neuromuscular condition	H49.81*, M12.0*, M36.0, E75.02, E75.19, E75.4, F71., F72., F73., F84.2, G10., G11.*, G12.*, G13.*, G14., G20.*, G21.*, G23.*, G24.*, G25.*, G26., G31.*, G32.*, G35., G36.*, G37.*,G40.*, G45.*, G46.*, G60.*, G61.*, G62.*, G63., G64., G70.*, G71.*, G73.*, G80.*, G81.*, G82.*, G83.*, G90.3, G91.*, G93.*, G94., G95.*, G99.2, P91.*, Q00.*, Q01.*, Q02., Q03.*, Q04.*, Q05.*, Q06.*, Q07.*, Q76.*, Q77.*, Q78.*, Q79.1, Q79.2, Q79.3, Q79.4, Q79.5*, Q79.6*, Q79.8, Q79.9, Q85.*, Q87.4*, Q91.*, Q92.*, Q93.*, Q96.*, R41.*, R53.2, R54.
Down syndrome	Q90.*

© 2026 Wiegand RE et al. *JAMA Network Open*.

Dystrophy	G71.0*
Pulmonary	
Asthma	J45.*
Chronic obstructive pulmonary disease	J40., J41.*, J42., J43.*, J44.*
Other lung disorders	D86.0, E88.01, J47.*, J60, J61, J62.*, J63.*, J64, J65, J66.*, J67.0, J67.1, J67.2, J67.3, J67.4, J67.5, J67.6, J67.7, J67.8, J68.*, J70.*,J81.1, J84.*, J95*, J96.1*, J99., P26.*, P27.*, B39.*, B40.1, B40.2, B41.0, B44.0, B44.1, B45.*, B46.0, A15.*, A31.0
Cystic fibrosis	E84.*
Renal	I12.*, I13.* N01.*, N02.*, N03.*, N04.*, N05.*, N06.*, N07.*, N08., N11.*, N14.*, N15.*, N16., N18.*, N25.*, N26.*, N28.*, Q27.1, Q27.2, Q60.*, Z49.*, Z91.15*, Z94.0, Z99.2
**Immunocompromising Conditions**	
Hematological malignancy	C81.*, C82.*, C83.*, C84.*, C85.*, C86.*, C88.*, C90.*, C91.*, C92.*, C93.*, C94.*, C95.*, C96.*, D46.*, D61.0*, D70.0, D61.2, D61.9, D71.*
Solid organ malignancy	C00.*, C01.*, C02.*, C03.*, C04.*, C05.*, C06.*, C07.*, C08.*, C09.*, C10.*, C11.*, C12.*, C13.*, C14.*, C15.*, C16.*, C17.*, C18.*, C19.*, C20.*, C21.*, C22.*, C23.*, C24.*, C25.*, C26.*, C30.*, C31.*, C32.*, C33.*, C34.*, C37, C38.*, C39.*, C40.*, C41.*, C43.*, C45.*, C46.*, C47.*, C48.*, C49.*, C50.*, C51.*, C52, C53.*, C54.*, C55, C56.*, C57.*, C58, C60.*, C61, C62.*, C63.*, C64.*, C65.*, C66.*, C67.*, C68.*, C69.*, C70.*, C71.*, C72.*, C73, C74.*, C75.*, C76.*, C77.*, C78.*, C79.*, C7A.*, C7B.*, C80.*,Z51.0, Z51.1*, C4A.*
Transplant recipient	T86.0*, T86.1*, T86.2*, T86.3*, T86.4*, T86.5, T86.81*, T86.85*, D47.Z1, Z48.2*, Z94.*, Z98.85
Rheumatologic/inflammatory disorder	D86.*, E85.1, E85.2, E85.3, E85.4, E85.8*, E85.9, G35.A, G35.B*, G35.C*, G35.D, J67.9, L40.54, L40.59, L93.0, L93.2, L94.*, M05.*, M06.*, M07.*, M08.*, M30.*, M31.3*, M31.5, M32.*, M33.*, M34.*, M35.3, M35.89, M35.9, M46.0*, M46.1, M46.8*, M46.9*
HIV/AIDS	B20, B97.35, O98.7*, Z21, and E88.14
Other intrinsic immune condition or immunodeficiency	D27.9, D72.89, D80.*, D81.0, D81.1, D81.2, D81.4, D81.5, D81.6, D81.7, D81.8*, D81.9, D82.*, D83.*, D84.*, D89.0, D89.1, D89.3, D89.4*, D89.8*, D89.9, K70.3*, K70.4*, K72.*, K74.3, K74.4, K74.5, K74.6*, N04.*, R18.0

*Includes all sub-codes.

© 2026 Wiegand RE et al. *JAMA Network Open*.
