# Supplementary material for: Interim Estimated Effectiveness of 2025-2026 COVID-19 Vaccines in Adults Using a Test-Negative Design
Source: JAMA Netw Open. 2026 Jun 23;9(6):e2625152. doi: 10.1001/jamanetworkopen.2026.25152 (PMC13291846; doi:10.1001/jamanetworkopen.2026.25152)
Supplement: Supplement 2. — Data Sharing Statement [file jamanetwopen-e2625152-s002.pdf]

Data Sharing Statement

Wiegand. Interim Estimated Effectiveness of 2025-2026 COVID-19 Vaccines in Adults. *JAMA Netw Open*. Published June 23, 2026. doi:10.1001/jamanetworkopen.2026.25152

Data
Data available: No

Additional Information
Explanation for why data not available: Data collected for the study are not available. Data sharing agreements between the CDC and VISION partner institutions prohibit the CDC from making this dataset publicly available.
